# When methods matter: solvent effects on pollinator responses to quantum-dot-labeled pollen

**DOI:** 10.1186/s13007-026-01544-3

**Published:** 2026-05-22

**Authors:** Barbara Płaskonka, Marcin Zych, Alina Liersch, Joanna Wolko, Maria Woszczenko, Katarzyna Roguz

**Affiliations:** 1 Botanic Garden, Faculty of Biology, University of Warsaw, Aleje Ujazdowskie 4, 00-478 Warsaw, Poland; 2 Plant Breeding and Acclimatization Institute—National Research Institute in Radzików,, Research Division in Poznań, Department of Oilseed Crops, Strzeszyńska 36, 60-479 Poznań, Poland; 3 Department of Biology, University of Marburg, 35042 Marburg, Germany

**Keywords:** *Brassica napus*, Hexane, Pollen transfer, Pollinator, Quantum dots, Solvent

## Abstract

**Background:**

Pollination, one of the key plant–animal interactions, has been intensively studied, yet a major gap remains in our ability to track the fate of individual pollen grains. Since 2019, the use of quantum dots (QDs) as an easy, cost-effective in situ pollen-labeling technique has begun to fill this gap by allowing direct, fine-scale tracking of pollen without impeding its transport. QDs are typically applied in a volatile organic solvent, a methodological step that is usually assumed to be behaviorally neutral, but could in principle alter floral visual and olfactory cues. Because accurate pollen tracking requires labeling methods that do not themselves affect pollinator behavior, we ask whether and how insect pollinators respond to flowers whose anthers have been treated with QDs and their solvent carrier.

**Results:**

Using a common-garden experiment with genetically uniform *Brassica napus* plants, we compared pollinator visitation and foraging behavior among flowers bearing QDs-solvent treatments, solvent-only controls, and untreated controls. Overall, our results support the use of QDs as a powerful and generally acceptable tool for tracking pollen transfer: pollinators did not avoid flowers with QDs-labeled pollen. Visitation did not differ among flowers labeled with different QDs colors, supporting within-study comparisons of multiple QDs colors. However, we detected an effect of the solvent: flowers exposed to QDs-solvent and solvent-only treatments received fewer visits and, in some cases, reduced foraging activity, particularly in honeybees.

**Conclusions:**

Because accurate pollen tracking requires labeling methods that do not themselves affect pollinator behavior, our findings show that quantum dots meet this requirement. However, the solvent used for their application can influence pollinator behavior. These effects, although moderate, indicate that potential side effects of solvents and labeling procedures should be carefully considered, controlled, and reported when designing QDs-based experiments and interpreting pollinator behavior.

**Supplementary Information:**

The online version contains supplementary material available at 10.1186/s13007-026-01544-3.

## Background

Pollination is one of the most important plant–animal interactions driving the diversification and evolution of seed plants and animals [73]. Two centuries of studies of plant–pollinator interactions have brought us closer to understanding the forces and mechanisms that shape them. Early work on pollination was largely descriptive, focusing on floral mechanisms and natural history [48, 62, 69] and was later complemented by an explicitly evolutionary perspective [11–13]. Together, these strands laid the foundations of modern pollination ecology [46].

Despite remarkable advances in the field, a persistent gap remains in our understanding of pollen movement [1, 41, 44], owing to the limited ability to track individual pollen grains [43]. While affordable and accurate, paternity analyses allow identification of which plants sired which seeds [25]. Minnar et al. [44, 45] correctly point out that, although informative, these methods cannot deliver a complete understanding of floral function, evolution, and ecology, as it requires tracking all pollen grains, not only those that successfully fertilized ovules. This field seems especially interesting, as only a small fraction of dispersed pollen ever reaches a stigma, on the order of ~ 2%, with the remaining ~ 98% lost en route [79]. Such massive attrition can be potentially related to selection on traits that govern pollen pickup, retention, and deposition, yet this field has remained poorly quantified.

Most studies are unable to link post-fertilisation measures of mating success to selective agents that act on traits that facilitate pollen transport [43]. Furthermore, the lack of suitable techniques to track pollen has limited our understanding of the actual importance of specific pollinators for plant reproduction, because visit frequency is only a proxy for pollen transfer efficacy [30, 78]. Determining the ecological importance of particular pollinators for different plant species is crucial for understanding species biology and evolution, and for designing effective restoration of species threatened with extinction. This matters especially when we realize the scale of dependence of the plant-pollinator interactions. Animal pollination supports ~ 87% of wild plant species and ~ 70% of crop species globally [31, 52], so questions about pollen transfer are also central to the economy, food security, and ecosystem functioning.

Despite its importance, quantifying male reproductive success remains challenging. Exceptions include taxa such as Orchidaceae and Asclepiadaceae, where pollen is packaged into pollinaria. Most angiosperms produce granular pollen that is difficult to track [3, 43]. Traditional approaches such as radioactive tracers [10] or fluorescent dyes [60, 71, 74] raise concerns about environmental exposure to radioactive labels and about the accuracy of the resulting inferences [21, 74, 76].

To this end, Minnaar and Anderson [43] published the first description of using quantum dots (QDs) as an easy, cost-effective, in situ pollen-labeling method. QDs are semiconductor nanoparticles (2–10 nm in diameter [49]) that emit bright visible light when excited with UV radiation [7, 18, 19]. They can attach directly to pollen and appear not to impede transport [43]. Quantum dots have been increasingly used to study pollen movement across ecological scales [27].

Protocols for QDs pollen labeling typically include suspending QDs in a volatile, nonpolar organic solvent (often hexane) that is applied directly to anthers; the solvent evaporates rapidly, leaving QDs attached to the pollen grains and has so far been assumed to be behaviorally neutral to pollinators [44]. Yet such solvents are widely used to extract floral volatiles and other lipophilic metabolites from flowers and pollen, indicating that they can strongly affect the composition of emitted scent bouquets (e.g., [32, 40]).

Applications of quantum dots now span multiple ecological scales. At the within-population scale, QDs have been used to trace fine-grained pollen movement [45]. Building on this, Minnaar and Anderson [42] examined pollen movement in the handed flowers of *Wachendorfia paniculata*, using floral asymmetry to test how organ positioning shapes pollen deposition. At the pollinator-performance scale, QDs have been used to quantify pollen-transfer efficacy in bees foraging among Asteraceae inflorescences [33], and to map pollen placement in bimodal floral tubes of *Lapeirousia anceps*, revealing how different floral morphologies load pollen onto a long-proboscid fly [45]. The method has also been applied in urban systems to compare pollen flow across seasons and pollinator groups [57–59]. Beyond movement per se, QDs have revealed pollen layering and male–male competition: sequential sampling from pollinator bodies showed that recently deposited labeled pollen is overrepresented in upper layers [47]. In addition, work in *Hypenia macrantha* suggests that explosive pollen placement may confer a pre-pollination male-competition advantage [2]. Despite this rapid uptake across systems, previous studies have focused on tracking performance and have largely assumed that the solvent and labeling procedure do not themselves alter pollinator behavior. To our knowledge, no study has explicitly disentangled the effects of QDs from those of their solvent carrier on pollinator visitation and foraging activity.

Given the centrality of accurate pollen tracking for linking visitation to effective pollen transfer, and thus to fruit/seed set and demography, QDs offer a practical path forward. However, any labeling method must be behaviorally neutral. In QDs protocols, the labeling step always combines two components (1) the QDs themselves and (2) the volatile organic solvent used to apply them; and either could modify floral visual or olfactory cues. Therefore we ask: how do insect pollinators perceive flowers whose anthers have been treated with QDs?

Pollen-eating insects can orient to pollen and/or anthers using olfactory [14, 37, 55], gustatory [22, 67, 75], and visual [38]. We therefore consider two communication channels that QDs could plausibly affect: visual and olfactory. Visually, attraction depends on floral shape and size but, for most visitors, is driven above all by color and color patterns [77]. In many species, pollen functions not only as the male gamete carrier and a food reward, but also as a visual signal [36, 63]. Yellow pollen is especially attractive to bees—many species are drawn to these cues and may even require visual pollen stimuli to initiate foraging [16].

In the second case, floral volatiles act as long-distance attractants and provide species-specific chemical signatures that influence pollinator preference and constancy [55]. Any changes in the olfactory cues emitted by pollen or anthers can also potentially influence plant-pollinator interaction. Because many floral volatiles and pollen-coat lipids are readily soluble in nonpolar organic solvents, even brief solvent exposure could temporarily deplete or redistribute these compounds, subtly altering the scent and taste landscape experienced by foraging insects [32].

Accordingly, we test whether QDs treatment of anthers alters pollinator visitation and behavior. We conducted a common-garden experiment using genetically uniform *Brassica* plants (to minimize within-species variation) and recorded pollinator responses to four treatments. To determine the potential effects of pollen labeling with QDs on shaping plant-pollinator interaction, we designed an experimental plant population, where we offered pollinators rapeseed plants (*Brassica napus*) with (1–3) flowers treated with three types of QDs + solvent, (4) solvent, (5) control, unlabeled pollen. This design allows us to separate the behavioral effects of QDs from those of the solvent and to assess whether the full labeling procedure can be considered behaviorally neutral.

We hypothesized that:

1. Solvent exposure may reduce pollinator visitation and foraging activity;
2. QDs + solvent flowers are expected to show similar pollinator responses to solvent-only flowers, indicating that QDs themselves do not affect pollinator behavior.

## Materials and methods

### Studied species

*Brassica napus* L. (Brassicaceae) is one of the most important mass-flowering crops, cultivated widely across Europe, USA, Canada, Brazil and China [20, 70]. It is partially wind pollinated, however its open, bright-yellow, and easily accessible flowers, offering a high volume of sugar-rich nectar, and sticky pollen favor insect pollination [53]. Rapeseed is visited by a wide range of pollinators including honeybees, bumblebees, solitary bees and hoverflies [70]. To reduce the impact of intraspecific diversity of plants (e.g., intensity of scent emitted and/or chemical composition of nectar) on attractiveness to pollinators, the experiment was conducted on genetically identical open-pollinated winter rapeseed cultivar Monolit.

### Plant material

The plant material used in this study was the genetically uniform open-pollinated cultivar ‘00’ of winter oilseed rape (cv. Monolit), characterized by an erucic acid content of up to 2% and a glucosinolate content of up to 15 μmol·g⁻^1^ seeds. Seeds were provided by the Department of Oilseed Crops of the Plant Breeding and Acclimatization Institute—National Research Institute, Poznań, Poland. In December 2024, seeds were sown in universal soil in 0.3 L pots. The pots were exposed to light for 12 h a day (the illuminance was 6000 lx) and kept at 17 °C (SD ± 0.6 °C) in the glasshouse. At the 4–5 leaf stage, seedlings were vernalized at 7 °C (SD ± 0.6 °C) for 9 weeks. After vernalization, plants were re-potted into 7 L pots. Post-vernalization plants were grown at a temperature of 20 °C (SD ± 0.7 °C) under supplemental illumination (7200 lx) to stimulate the flowering process and supplied with nutrients using Florovit fertilizer. After the first flower buds appeared, the plants were transferred outside, placed at equal distances from each other, and watered regularly until they fully bloomed. All experimental work was conducted at the University of Warsaw Botanic Garden, Warsaw, Poland.

### Experimental setup

To evaluate the effects of using QDs on pollinator activity, plants grown in the controlled conditions (Supplementary materials S1) were arranged in five experimental treatments: (1) control, plants with unlabeled pollen; (2) plants with pollen labeled with hexane only; (3–5) plants with pollen labeled with a solution of hexane and QDs inducing radiation of three lengths (hereafter: QDs-hexane solution). Two QDs of the available extreme wavelength (530 nm, emitted color when exposed to UV light: yellow; 700 nm, emitted color when exposed to UV light: red) and one with an intermediate wavelength (590 nm, emitted color when exposed to UV light: orange) were selected. Rapeseed pollen lacks typical pollenkitt, instead having tryphine [43]. However, under the microscope with UV light, we confirmed that QDs adhered well and were clearly visible on the pollen grains.

**Fig. 1:**
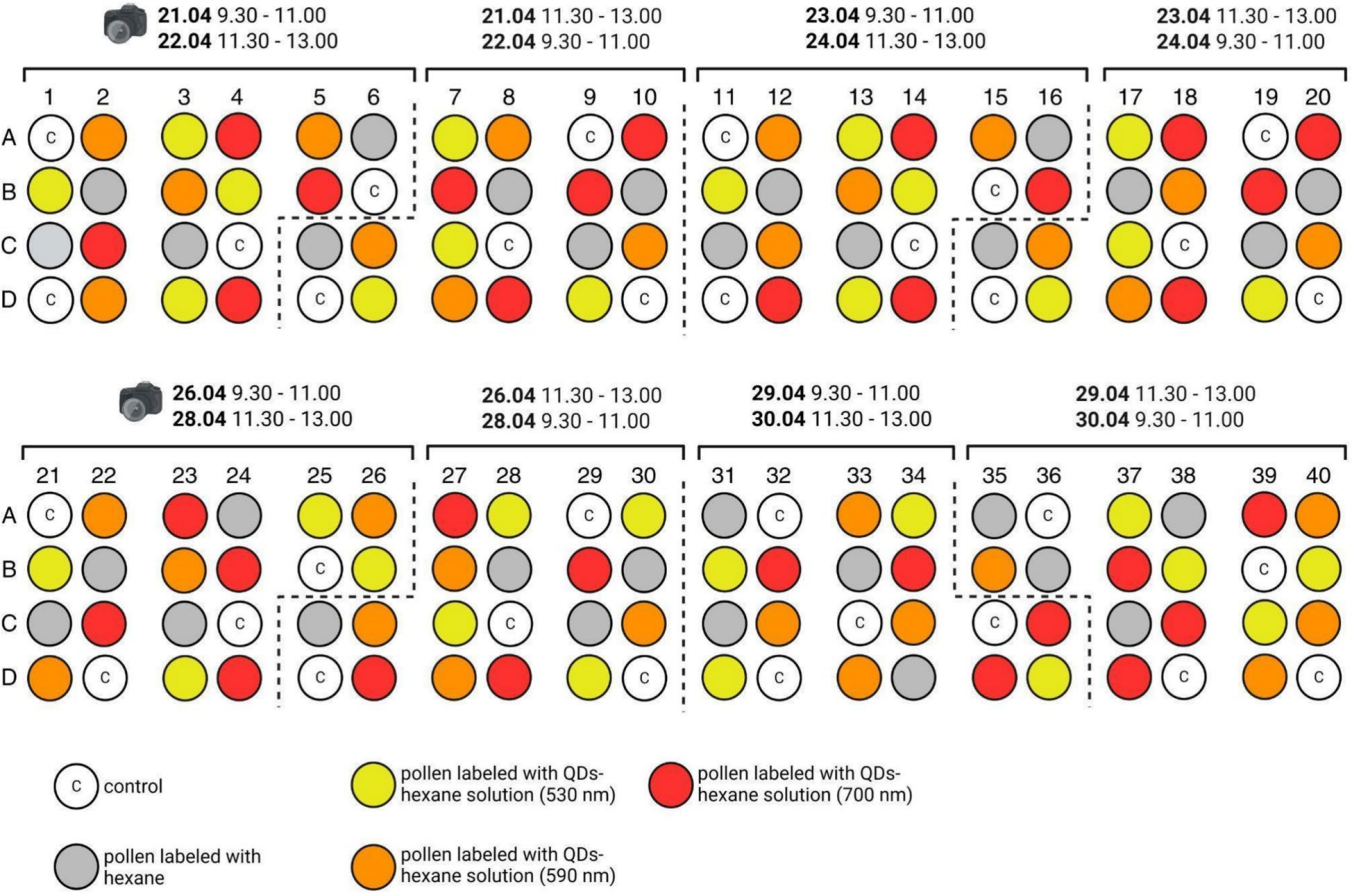
Experimental scheme. Plants are represented by circles with colors corresponding to experimental treatments. Dates and recording times are indicated above each group of 20 plants

**Fig. 2:**
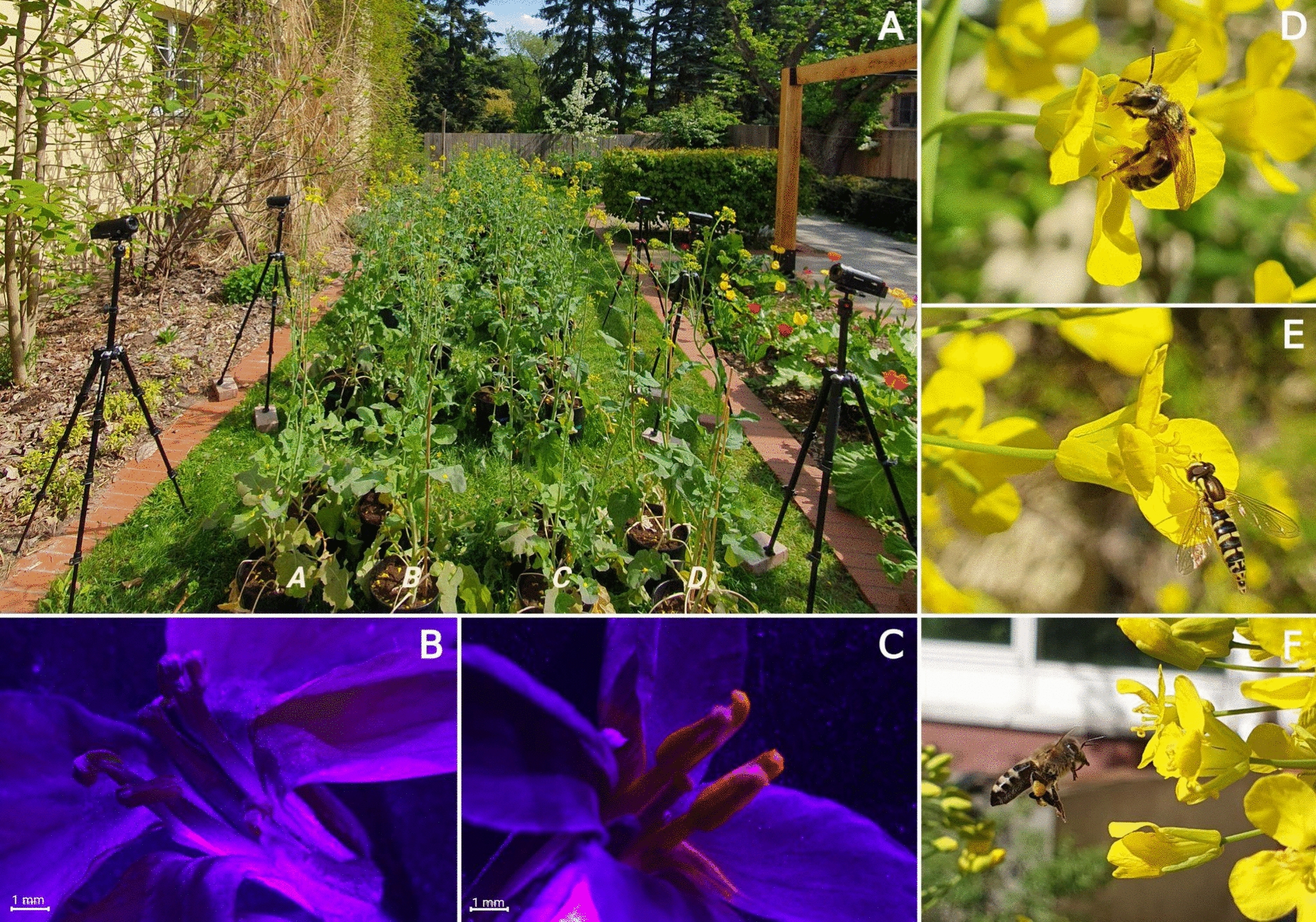
**A** The experimental setup showing 20 rows of plants arranged in a grid (4 pots per row). Letters **A**–**D** indicate consecutive columns of plants. Each block consisted of 20 plants, which were simultaneously recorded using five digital cameras. Oilseed rape flowers with **B** control anthers and **C** anthers labeled with QDs (590 nm) under the UV light. Pollinators observed foraging on oilseed rape flowers: **D** solitary bee (*Andrena* sp.), **E** Syrphidae (*Sphaerophoria* sp.), and **F** *Apis mellifera.*

Pots (n = 160; arranged in 20 rows of 4 pots each, equally spaced, across two rounds) were assigned to experimental treatments at random with two constraints: no individuals per treatment were adjacent, and each block of 20 pots contained equal representation of individuals per treatments (as indicated by the dotted lines in Fig. 1). After testing pollinator activity on the first set of 80 plants, they were replaced by a second set of 80 pots (Fig. 2A).

The study was conducted in 2025 under field conditions over eight days during good weather (no rain or strong wind) between April 21 and April 30. To assess pollinator activity on rapeseed flowers with unlabeled and labeled pollen, hexane or a QDs-hexane solution were applied to the designated plant variants (Fig. 2B, C). All the anthers of the newly opened flowers were labeled early each morning (08:00–09:00), with approximately 3 μL of solution applied per flower using automatic pipette. Old flowers were removed to prevent bias associated with differences in the ratio of newly opened to older flowers on each plant. Pollinator activity was observed between 09:30 and 13:00 using digital cameras (Panasonic HC-VX980 or HC-V380; Fig. 2A). Each plant was recorded on two consecutive days for 1.5 h/day, resulting in a total sampling effort of 480 h (160 plants × 2 days × 1.5 h). During each 1.5 h interval, 20 plants were recorded and then the cameras were moved to the next 20. On the second day, the order was reversed to balance time-of-day effects. Forty plants were surveyed on two consecutive days. In the laboratory, videos were reviewed for frequency of pollinator visits, time spent on the flower and the number of visited flowers. Visit frequency (visits/h) was computed as the number of visits per 90-min recording multiplied by 2/3. Visitors were assigned to morphogroups: honeybees (*Apis mellifera*), solitary bees, bumblebees (*Bombus*), hoverflies (Syrphidae), muscoid flies (Muscoidea), and butterflies (Fig. 2D–F).

### Solvent (hexane) tests

To test short-term changes in pollen mass following exposure to hexane, five replicates were prepared with all six anthers from each of five flowers at anthesis (30 anthers per sample) extracted and transferred, using clean forceps, to pre-weighed plastic boats. Initial mass was recorded on an analytical balance (Kern ALJ 210-5A). Then, 10 µL of hexane was pipetted evenly over the anthers. Subsequent mass was recorded at 1, 3, 5, and 10 min after hexane application, with the sample remaining on the balance between readings. Measurements were performed in five repetitions on five independent samples.

To assess the effect of hexane treatment on the optical properties of anthers, their reflectance across the visible spectrum (350–700 nm) was measured. Anthers in the anthesis were extracted from several dozen flowers and pooled so that they covered a continuous layer of approximately 1 cm^2^. The second group of samples was prepared the same way and treated with 30 µL of hexane. Reflectance of two control samples and two samples treated with hexane was measured using a Jaz spectrometer (Ocean Optics).

### Statistical analysis

To evaluate the effects of the treatment, the number of flowers per plant, and the time of day on the frequency of pollinator visits, visit duration, and number of visited flowers generalized linear mixed models (GLMMs) were used. Analyses were conducted for all pollinators combined and separately for the two most common groups (honeybees and solitary bees). Random effects for plants nested within blocks within dates (for visit frequency and number of visited flowers) or plants nested within dates within visited flowers (for visit duration) were included to account for the hierarchical structure of the data.

For visit frequency, zero-inflated negative binomial GLMMs using the ‘glmmTMB’ function in the ‘glmmTMB’ package [6] was applied, as it prevented overdispersion. For the visit duration and the number of visited flowers, standard negative binomial GLMMs were used. Model selection was based on minimizing the Akaike information criterion (AIC) using the MuMIn::dredge() function [4]. The final model was compared with a reduced model (excluding the treatment effect) using ANOVA to test whether treatment had a significant effect.

To illustrate differences among treatments, estimated marginal means (EMMs) were calculated using the emmeans::emmeans() function [34] and model predictions were visualized as marginal responses, i.e., predicted values assuming all other predictors at a constant (mean) level using the ggeffects::ggpredict() function [35].

To assess how anthers treated with hexane are perceived by honeybees, reflectance spectra (350–700 nm) were analysed and processed in the pavo package [39]. Honeybee color perception was modeled using vismodel(), and hexagon color space coordinates were calculated with colspace() [8], allowing comparison of color differences between samples. All analyses were conducted in R version 4.1.2 [61].

## Results

We recorded a total of 8936 pollinator visits to *B. napus* plants, six morphogroups: *Apis mellifera* (6791 visits; 76% of all visits), solitary bees (2049; 23%), *Bombus* (73; 0.8%), Syrphidae (28; 0.3%), butterflies (11; 0.1%) and Muscoidea (9; 0.1%).

**Fig. 3:**
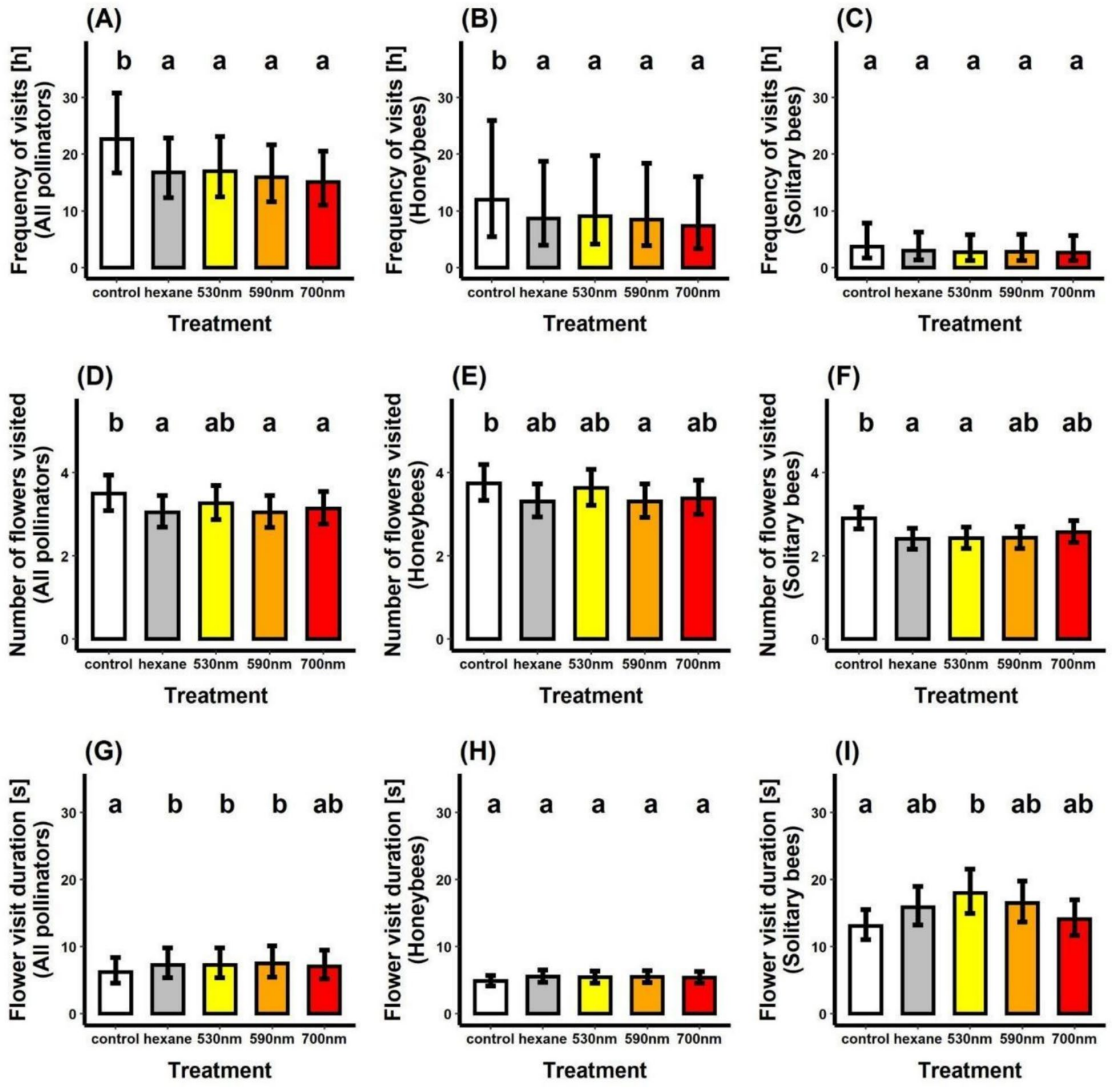
Effects of anther treatments on pollinator activity. The columns show different pollinator groups: all pollinators, and the two most frequent pollinator groups, i.e., honeybees and solitary bees. The rows show different response variables: **A**–**C** frequency of visits/h, **D**–**F** number of flowers visited during a single foraging bout, and **G**–**I** duration of a single flower visit (s). Different letters above bars indicate statistically significant differences between treatments

For all recorded pollinators, treatment had an effect on the frequency of visits (χ^2^ = 40.53, df = 4, *p* < 0.01; Fig. 3A). Plants in the control group were visited more often (21.2 ± 3.4 visits per hour) than flowers exposed to hexane or its solution with QDs (ranging from 14.1 ± 2.3 (700 nm) to 15.9 ± 2.5 visits per hour (530 nm)). All non-control variants did not differ.

The frequency of *A. mellifera* visits differed among treatments (χ^2^ = 34.3, df = 4, *p* < 0.01; Fig. 3B). Plants in the control group were visited more often (10.9 ± 4.4 visits per hour) than flowers exposed to hexane or its solution with QDs (ranging from 6.8 ± 2.7 (700 nm) to 8.4 ± 3.4 visits per hour (530 nm)). All non-control variants did not differ. In the case of solitary bees visits, the frequency of visits did not differ among treatments (χ^2^ = 9.3, df = 4, *p* = 0.054; Fig. 3C).

For all recorded pollinators combined, and in separate analyses for *A. mellifera* and solitary bees, both time of day (*p* < 0.01) and the number of flowers per plant (*p* < 0.01) positively influenced visitation frequency (Supplementary Fig. 1A–F).

For all pollinators, treatment had an effect on the number of flowers visited (χ^2^ = 19.04, df = 4, *p* < 0.01; Fig. 3D).

During a single visit to a plant, pollinators visited more flowers in the control plant (3.50 ± 0.22) than in the 590 nm, 700 nm, and hexane treatments (ranging from 3.04 ± 0.20 (590 nm) to 3.14 ± 0.20 (700 nm)). The 530 nm treatment (3.26 ± 0.21) did not differ from either the control or the other treatments.

In the case of *A. mellifera,* the number of flowers visited differed among treatments (χ^2^ = 13.16, df = 4, *p* = 0.01; Fig. 3E). Honeybees visited more flowers in the control treatment (3.74 ± 0.22) than in the 590 nm treatment (3.31 ± 0.20). The 530 nm, 700 nm, and hexane treatments (ranging from 3.31 ± 0.20 (hexane) to 3.63 ± 0.22 (530 nm)) did not differ from either the control or the 590 nm treatment.

In the case of solitary bees, the number of flowers visited differed among treatments (χ^2^ = 14.47, df = 4, *p* < 0.01; Fig. 3F). Solitary bees visited more flowers in the control treatment (2.89 ± 0.14) than in the 530 nm and hexane treatments (2.42 ± 0.13 and 2.40 ± 0.13, respectively). The 590 nm and 700 nm treatments did not differ from other treatments (2.41 ± 0.13 and 2.58 ± 0.13).

In the case of all pollinators, *A. mellifera* and solitary bee visits, both time of day (*p* < 0.01) and the number of flowers per plant (*p* < 0.01) positively influenced the number of flowers visited by pollinator (Supplementary Fig. 2A–F).

For all pollinators, treatment had an effect on the flower visit duration (χ^2^ = 18.94, df = 4, *p* < 0.001; Fig. 3G). The longest visits occurred in the 530 nm, 590 nm, and hexane treatments (7.27 ± 1.13, 7.47 ± 1.16, and 7.26 ± 1.13 s), while the shortest visits were recorded for the control treatment (6.19 ± 0.96 s). The 700 nm treatment did not differ from either the control or the 530 nm, 590 nm, and hexane treatments (7.02 ± 1.09 s).

In the case of honeybees visit duration differed slightly among treatments (χ^2^ = 9.50, df = 4, *p* = 0.05; Fig. 3H), with the shortest visits recorded for control flowers (4.88 ± 0.40 s) and marginally longer visits for all other treatments (ranging from 5.39 ± 0.47 (700 nm) to 5.55 ± 0.46 s (hexane)).

In the case of solitary bees, visit duration differed among treatments (χ^2^ = 12.04, df = 4, *p* = 0.017; Fig. 3I). The longest visits occurred in the 530 nm treatment (17.97 ± 1.68 s), while the shortest visits were recorded for the control treatment (13.10 ± 1.13 s). The 590 nm, 700 nm, and hexane treatments showed intermediate visit durations (ranging from 14.10 ± 1.34 (700 nm) to 16.47 ± 1.54 s (590 nm)).

In the case of all pollinators, honeybees and solitary bees, time of day (*p* < 0.01) negatively influenced the flower visit duration (Supplementary 3A–C), while the number of flowers per plant had no effect.

### Solvent (hexane) tests

Applying hexane did not influence the pollen mass. Immediately after hexane application, the measured mass exhibited a transient increase, consistent with residual solvent on the sample. By the third minute, the solvent had evaporated, and the measurements returned to baseline, remaining stable at the five- and ten-minute intervals.

Anthers labeled with hexane reflected less light than control anthers across almost the entire measured wavelength range (450–700 nm; Supplementary Fig. 4). The difference was particularly pronounced in the spectra between 550 and 680 nm.

Bee visual modeling showed that anther reflectance was dominated by the L (green, long-wavelength) receptor, with the following ranking of chromatic visibility: hexane_2 > hexane_1 > control_2 > control_1. The S (UV, short-wavelength) and M (blue, medium-wavelength) receptor responses were minimal, and the achromatic (luminosity) channel was not fully captured in these measurements (Supplementary Table S2).

## Discussion

In this study, we found that the use of hexane and QDs-hexane solution for labeling *B. napus* pollen affected pollinator behavior, including visit frequency, number of flowers visited per plant visit, and flower visit duration. While QDs are a powerful and promising tool for tracking pollen transfer [43], our results show that their application should be carefully considered, as the method used to apply them may affect pollinator behavior.

Honeybees visited flowers less frequently when pollen was marked with hexane or a QDs-hexane solution, suggesting that the solvent itself may influence how pollinators perceive flowers. The number of flowers visited during a single visit was also lower in the hexane and QDs-treated variants than in the control. For solitary bees, the duration of visits was longer on flowers treated with hexane and QDs when compared to the control flowers. The similarity of pollinator responses to hexane and QDs indicates that the latter themselves did not cause additional strong changes, and that the observed effects were mainly driven by the solvent.

The observed results may be caused by pollen properties. One of the functions of the pollen coat is to protect pollen from excessive water loss, although its role mainly consists in slowing down the drying process rather than preventing it completely [54]. When staining pollen with hexane or a solution of hexane and QDs, we observed that the anthers appeared dry, but weight measurements did not show any actual water loss. Nevertheless, there are other potential factors that could explain the observed effect of the hexane used and we assume they are related to plant-pollinator communication and preferences of the latter.

First, hexane may affect the light reflectance properties of anthers. By altering the surface structure of pollen grains, e.g., through partial dissolution of epicuticular waxes or carotenoids, it may modify how light, including UV radiation, is reflected from pollen surfaces [14, 26, 72]. In our study, anthers labeled with hexane reflected less light than control anthers, with the largest difference in the wavelengths between 550 and 680 nm, corresponding to the peak sensitivity of honeybee green photoreceptors [23]. The overall shape of the reflectance spectra remained similar, indicating that only brightness of the anthers, not color, was affected. Although hexane-treated anthers showed higher reflectance in the long-wave (green) region, bees did not preferentially visit them. Different pollinator groups, like birds and mammals, may perceive these changes differently depending on their visual systems, and therefore respond differently to the modified anther signals. At least for honeybees, the observed differences in brightness should not cause changes in plant-pollinator interaction, as discovered by Ng et al. [50], these pollinators do not use brightness cues in a color processing context. The visual ecology of solitary bee species remains unstudied, and it is unknown whether brightness cues affect their foraging behavior [68].

Second, hexane can change olfactory communication. Pollen, like other parts of the flower, emits its own characteristic scent, which differs in chemical composition from other flower parts. This scent originates primarily from the pollen coat—a lipidic layer surrounding the pollen grains [15, 24, 54]. Contact with an organic solvent such as hexane may alter the pollen scent profile by removing neutral lipids contained within the pollen coat, which are crucial for pollen odor [14]. Although the solvent evaporates rapidly, its action may temporarily disrupt the floral scent profile used by pollinators for flower recognition and choice [77]. Additionally, the possibility of residual solvent odor cannot be completely excluded. Even though mass measurements confirmed full evaporation of hexane, honeybees are capable of detecting volatile compounds at extremely low concentrations [5]. Thus, even trace amounts of unfamiliar substances could act as deterrent signals, leading bees to avoid "differently smelling" flowers.

Third, gustatory preferences may influence the potential changes in plant-pollinator interaction in hexane treated flowers. If the solvent even partially altered the taste of pollen, this could influence pollinators' perception of the reward after visiting the flower. Therefore, any of the three mentioned changes or the mixture of them may influence the way honeybees interact with visited flowers. These pollinators rapidly learn to associate specific colors or odors with a lack of reward or with negative stimulus [9, 66]. If individual foragers experienced that flowers treated with hexane offered a less attractive reward, they may have subsequently avoided similar flowers.

When comparing honeybees and solitary bees, differences can be observed in their responses to flowers treated with hexane and QDs. Honeybees showed a stronger avoidance of such flowers, whereas solitary bees appeared to compensate for potential sensory disturbances by extending their visit duration. In the case of solitary bees, no significant differences were found in visitation frequency among treatments, however, changes in the number of flowers visited and the duration of visits were noticeable. The lack of differences in visitation frequency suggests that solitary bees may be less sensitive to visual and olfactory changes than honeybees, or that they respond more individually, without social learning effects [56]. Nonetheless, the longer visits observed in solitary bees may reflect the need for a more thorough assessment of altered visual and olfactory stimuli associated with the floral reward.

Honeybees visited control flowers more frequently than flowers with labeled pollen, while the duration of individual visits did not differ significantly. Honeybees rely on precise memorization of visual and olfactory patterns and features associated with the reward [56, 77], whereas solitary bees have comparatively poorer learning abilities [17]. Moreover, social learning may play an important role, as honeybees are known to rapidly communicate information about resource quality [29].). If early foragers experienced negative cues (e.g., altered scent), they could have transmitted this information to other workers, leading to an overall decline in visitation frequency [51].

Despite the observed differences in visitation frequency, number of flowers visited, and visit duration, the overall effect of hexane and QDs on pollinator behavior was relatively modest. Pollinators, including both honeybees and solitary bees, did not avoid flowers whose pollen was labeled with QDs, indicating that the method of marking pollen with QDs can be applied in studies of pollen transport. Quantum dots are increasingly used in studies of pollen movement and pollination ecology [2, 28, 59, 64, 65], representing a powerful tool for tracking pollen transfer in the field.

However, our results clearly demonstrate that the solvent used for QDs application can influence pollinator behavior. Therefore, we strongly recommend including a solvent-only control in future studies to disentangle the effects of the labeling method from those of the solvent itself. The observed reduction in foraging activity, particularly among honeybees, suggests that potential side effects of such treatments should be taken into account when interpreting results from field experiments, especially those addressing pollinator preferences.

## Supplementary Information

Supplemantary material 1.

## Data Availability

The datasets generated and/or analysed during the current study are available in the Dane Badawcze UW repository, https://doi.org/10.58132/HV6AYG (Płaskonka, 2025).
