# Supplementary material for: When methods matter: solvent effects on pollinator responses to quantum-dot-labeled pollen
Source: Plant Methods. 2026 May 22;22:66. doi: 10.1186/s13007-026-01544-3 (PMC13455316; doi:10.1186/s13007-026-01544-3)
Supplement: Supplementary file 1 [file 13007_2026_1544_MOESM1_ESM.docx]

Supplementary Fig.1 Effects of [A-C] time of day and [D-F] number of flowers per plant on the frequency of visits of different pollinator groups: all pollinators, and the two most frequent pollinator groups, i.e., honeybees and solitary bees.

Supplementary Fig.2 Effects of [A-C] time of day and [D-F] number of flowers per plant on the number of flowers visited by different pollinator groups: all pollinators, and the two most frequent pollinator groups, i.e., honeybees and solitary bees.

Supplementary Fig. 3 Effects of time of day on the flower visit duration of different pollinator groups: [A] all pollinators and the two most frequent pollinator groups, i.e., [B] honeybees and [C] solitary bees.

Supplementary Fig. 4 [A] Reflectance and [B] bee visual modeling of control and hexane-treated anthers across 350–700 nm.

Table S2. Honeybee visual model outputs for all samples. S (UV), M (blue), and L (green) denote photoreceptor excitations; *x* and *y* are hexagon coordinates.

| Sample | S | M | L |
| --- | --- | --- | --- |
| hexane_1 | 0.000882611 | 0.011018315 | 0.10606814 |
| hexane_2 | 0.001307634 | 0.015482147 | 0.11982002 |
| control_1 | 0.000437024 | 0.005296622 | 0.06722292 |
| control_2 | 0.000522488 | 0.006481172 | 0.08172809 |
